# Restriction of Neural Precursor Ability to Respond to Nurr1 by Early Regional Specification

**DOI:** 10.1371/journal.pone.0051798

**Published:** 2012-12-11

**Authors:** Chiara Soldati, Emanuele Cacci, Stefano Biagioni, Nicoletta Carucci, Giuseppe Lupo, Carla Perrone-Capano, Isabella Saggio, Gabriella Augusti-Tocco

**Affiliations:** 1 Department of Biology and Biotechnology-Neurobiology Research Unit, and “Daniel Bovet” Neurobiology Research Center, “Sapienza” University of Rome, Rome, Italy; 2 “Istituto Pasteur Fondazione Cenci Bolognetti”, Sapienza University of Rome, Rome, Italy; 3 Department of Biological Sciences, University of Naples Federico II, Naples, Italy; 4 IGB “A. Buzzati Traverso”, Naples, Italy; 5 Department of Biology and Biotechnology, “Sapienza” University of Rome, Rome, Italy; 6 “Istituto Pasteur Fondazione Cenci Bolognetti”, “Sapienza” University of Rome, Rome, Italy; 7 Institute of Molecular Biology and Pathology, CNR, Rome, Italy; Baylor College of Medicine, United States of America

## Abstract

During neural development, spatially regulated expression of specific transcription factors is crucial for central nervous system (CNS) regionalization, generation of neural precursors (NPs) and subsequent differentiation of specific cell types within defined regions. A critical role in dopaminergic differentiation in the midbrain (MB) has been assigned to the transcription factor Nurr1. Nurr1 controls the expression of key genes involved in dopamine (DA) neurotransmission, e.g. tyrosine hydroxylase (TH) and the DA transporter (DAT), and promotes the dopaminergic phenotype in embryonic stem cells. We investigated whether cells derived from different areas of the mouse CNS could be directed to differentiate into dopaminergic neurons in vitro by forced expression of the transcription factor Nurr1. We show that Nurr1 overexpression can promote dopaminergic cell fate specification only in NPs obtained from E13.5 ganglionic eminence (GE) and MB, but not in NPs isolated from E13.5 cortex (CTX) and spinal cord (SC) or from the adult subventricular zone (SVZ). Confirming previous studies, we also show that Nurr1 overexpression can increase the generation of TH-positive neurons in mouse embryonic stem cells. These data show that Nurr1 ability to induce a dopaminergic phenotype becomes restricted during CNS development and is critically dependent on the region of NPs derivation. Our results suggest that the plasticity of NPs and their ability to activate a dopaminergic differentiation program in response to Nurr1 is regulated during early stages of neurogenesis, possibly through mechanisms controlling CNS regionalization.

## Introduction

During development, lineage commitment is a multistep process requiring the activation and repression of sets of genes at successive stages, leading from an embryonic stem (ES) to a tissue-specific stem cell identity as neural stem cells (NSCs). NSCs are capable of giving origin to glial cells and different populations of neurons. How and when the different phenotypic features that underlie cell diversity are specified is an important issue in developmental neuroscience. Cell fate specification appears to occur early during development and is governed by a complex combination of intrinsic and extrinsic factors. Positional identity is assigned to NSCs via gradients of signaling molecules secreted throughout the dorsoventral and rostro-caudal axes of the neural tube during defined temporal windows. Once neuronal fates have been specified and restricted by these extrinsic cues, intrinsic signals direct differentiation into mature neurons. [1], [2]. Despite major progresses in this field, the molecular mechanisms as well as the extrinsic and intrinsic factors underlying lineage commitment in NSCs remain only partially understood [3]–[5]. In this context, an important issue is to understand when positional identity is acquired by neural precursors (NPs) and whether it can be maintained when NPs are removed from their environment and cultured in vitro under defined experimental conditions.

Cells displaying properties of NPs have been isolated and cultured in vitro by exposing cells derived from different regions of the Central Nervous System (CNS) to growth factors, which maintain NPs in a proliferative and undifferentiated state; upon growth factor deprivation NPs can undergo differentiation and give rise to a mixed population of neurons and glial cells. Despite some contradictory data reported in previous studies, regional identity seems to be, at least partially, conserved after NP expansion *in vitro* [6]–[9]. Preservation of positional identity has recently been re-examined and shown to be maintained over 20 passages in vitro by NPs derived from 14.5 mouse cortex [10]. These findings raise the question of whether acquisition of positional identity may restrict the ability of NPs to respond to transcription factors that direct or cooperate to the acquisition of specific neuronal identities. A well-known example is represented by midbrain (MB) dopamine (DA)-producing neurons, whose specification requires the expression of a set of specific transcription factors [11]–[15]. Among these, Nurr1 has a critical importance for the differentiation and maintenance of MB DA neurons. In mice, high Nurr1 expression is detected at E10.5 in newly born postmitotic DA neurons [16], [17] and is maintained in the adult. Nurr1 controls the expression of key genes involved in DA neuron function such as tyrosine hydroxylase (TH), vesicular monoamine transporter 2, DA transporter (DAT), the co-receptor for the GDNF family Ret and the neurotrophin BDNF [17]–[19]. Nurr1 overexpression can positively influence the ability of embryonic stem (ES) cells and NPs to differentiate into DA neurons [20]–[23]. Previous reports on the ability of Nurr 1 to activate TH expression used short-term cultures of NPs [24] and only a few studies have investigated the ability of multipassaged NPs to generate dopaminergic neurons in vitro [22], [25]. However, the use of long-term expanded NP lines provides a significant advantage for establishing NPs properties and their possible use in transplantation experiments.

In this work, we have used growth factor-immortalized cells to test Nurr1's ability to activate dopaminergic differentiation in relation to the regional character of NPs. Our data show a differential ability to respond to Nurr1 overexpression of NPs from different CNS regions; this observation supports the existence of mechanisms that restrict NP plasticity, possibly due to the specification and maintenance of “positional identity” cues. To the best of our knowledge this is the first study comparing the ability of long-term expanded murine NP cell lines from different CNS areas to generate TH-positive neurons under uniform experimental conditions.

## Results

### Neural Precursors from embryo and adult CNS

**Figure 1:**
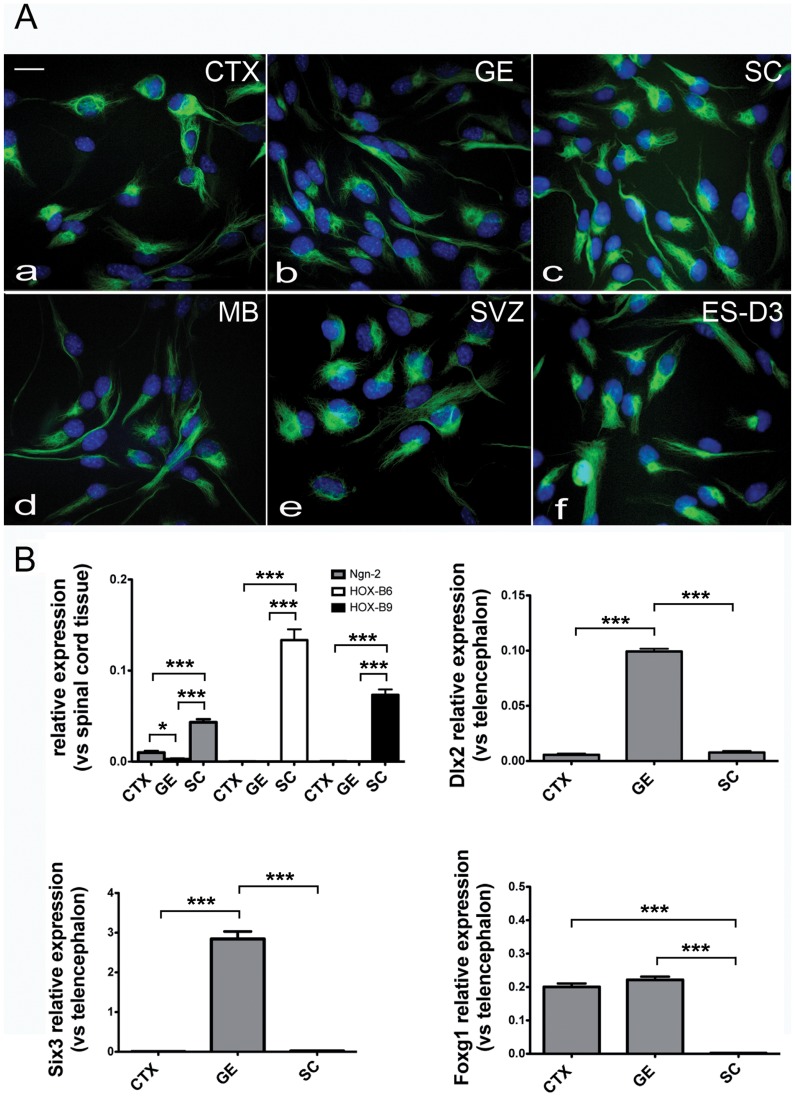
Immunodetection of Nestin in NPs lines derived from different brain regions and RT-PCR detection of regionally expressed genes. A) Immunostaining for nestin (green) on NPs derived from a) cortex (CTX), b) lateral ganglionic eminence (GE), c) spinal cord (SC), d) midbrain (MB), e) subventricular zone (SVZ), f) mouse blastocyst (ES-D3). Nuclei were stained with DAPI (blue). Scale bar  = 20 μm. B). B) Real time PCR quantification of Ngn2, HoxB6, HoxB9, Dlx2, Six3 and FoxG1 expression in CTX-, GE- and SC-derived NPs cultured in proliferating conditions. Results are shown as the mean of the ratio between gene expression in NPs and in dissected E13.5 tissue (telencephalon or spinal cord) in four biological replicates. Error bars show the standard error of the mean. * P<0,05 ***p<0.001.

Nurr1 ability to activate expression of dopaminergic markers was analyzed in NP lines adapted to in vitro cell culture as previously described [26]. NPs were isolated from various areas of the mouse E13.5 embryo CNS, namely cortex (CTX), ganglionic eminence (GE), spinal cord (SC), midbrain (MB), and from the adult subventricular zone (SVZ). Cells were propagated as adherent cultures in the presence of bFGF and EGF, as described in the Materials and Methods section. Nestin immunoreactivity was found in 100% of cells when NPs were cultured under proliferative conditions, confirming NP identity (Fig. 1A). NPs were also characterized following in vitro culture (6–7 passages) for the expression of molecular markers of the regions of derivation. In particular, FoxG1 is expressed in the developing mouse telencephalon (CTX and GE), Dlx2 and Six3 are expressed in the developing GE, HoxB6 and HoxB9 are expressed in the developing SC, while Ngn2 is expressed in both CTX and SC [7], [8], [10], [27]–[30]. Real-time PCR analysis showed that CTX-, GE- and SC-derived NPs maintain in vitro expression profiles coherent with the corresponding CNS regions (Fig. 1B). These expression profiles were reproducible in NP lines derived from independent dissection experiments (data not shown). These observations are consistent with a recent study showing that region-specific molecular signatures are preserved in NPs derived from different areas of the developing mouse CNS [10], indicating that positional identities are at least partially maintained by NPs used to test Nurr1 ability to promote dopaminergic cell fates.

**Figure 2:**
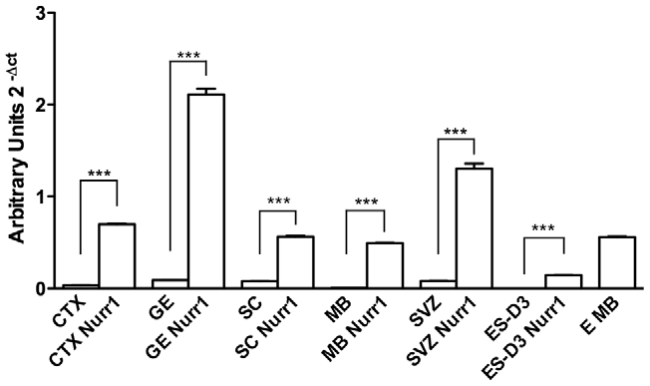
Nurr1 overexpression in NPs lines following lentivurs infection. Nurr1 mRNA levels detected by Real Time PCR in CTX, GE, SC, MB, adult SVZ and in ES-D3 derived NPs in control conditions and after infection with Nurr1 lentivirus. Nurr1 mRNA level in E12 mouse embryo MB (E MB) is shown as the positive control. The error bars represent standard deviation (n = 3). *** P<0,001.

**Figure 3:**
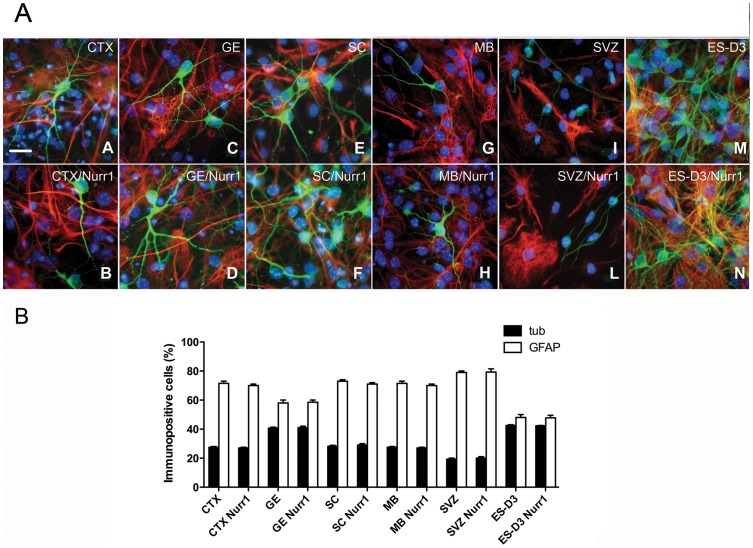
Evaluation of neuronal and glial cells in differentiated NPs lines. A) Immunostaining for beta-III-tubulin (green) GFAP (red) on differentiated cells in control conditions (A, C, E, G, I.M) and after infection with Nurr1 lentivirus (B, D, F, H, L, N). (A, B) CTX, (C, D) GE, (E, F) SC, (G, H) MB, (I, L) adult SVZ, (M, N) ES-D3. Nuclei were stained with DAPI. **B**) Percentage of beta-III-tubulin and GFAP positive cells after differentiation of NPs. The reported values are the mean ± SEM of 10 observations from two independent experiments Scale bar  = 20 μm. The error bars represent standard deviation.

As described in the Materials and Methods section, we either transiently infected NPs with Nurr1-expressing lentivirus or generated NP transgenic lines with stable Nurr1 expression following electroporation with a plasmid carrying Nurr1 and antibiotic selection. In either case, NPs expressing Green Fluorescent Protein were used as a control. Expression of Nurr1 was evaluated by Real-time PCR 72 hours after infection or in transgenic lines, showing that with both approaches Nurr1 mRNA levels were markedly increased in all NPs lines compared to control cells and reached levels comparable or even higher than those in E12 MB (Fig. 2 and Fig. S1). NP cells were then exposed to a differentiation protocol, as described in Materials and Methods and characterized by immunostaining and Real-timePCR. The presence of neuronal (beta-III-tubulin) and glial (GFAP) markers was evaluated in either controls or Nurr1-expressing cells after 7 days of differentiation (Fig. 3 A, B and Fig. S1). Although the extent of neuronal and glial differentiation was somewhat different among NPs derived from various CNS regions, ranging from 20% to 40% of beta-III-tubulin positive cells and 60–80% glia, Nurr1 overexpression did not change the percentage of beta-III-tubulin positive cells in NPs derived from various CNS regions.

**Figure 4:**
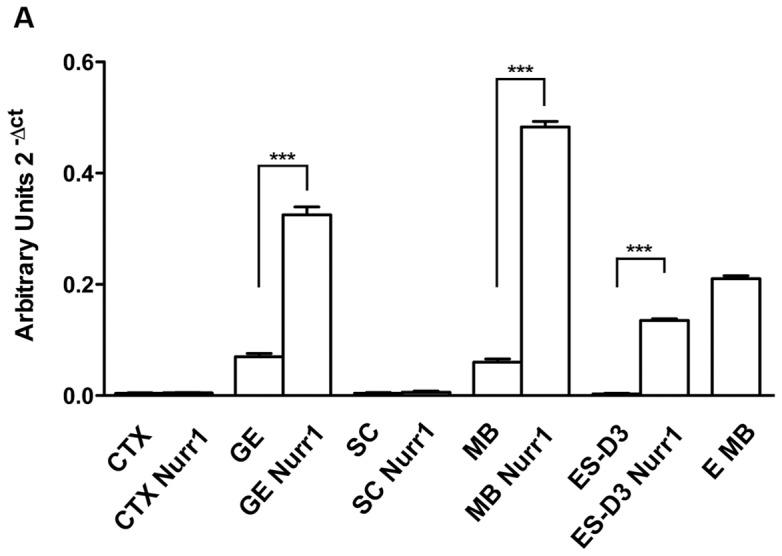
Induction of TH expression by Nurr1 overexpression: RT-PCR analysis. TH mRNA level detected by Real Time PCR in differentiated CTX, GE, MB and ES-D3 NPs and in cells infected with Nurr1. TH mRNA was not detectable in other analyzed NPs. TH mRNA level in mouse MB (E MB) is reported as positive control. The error bars represent standard deviation (n = 3). *** P<0,001.

**Figure 5:**
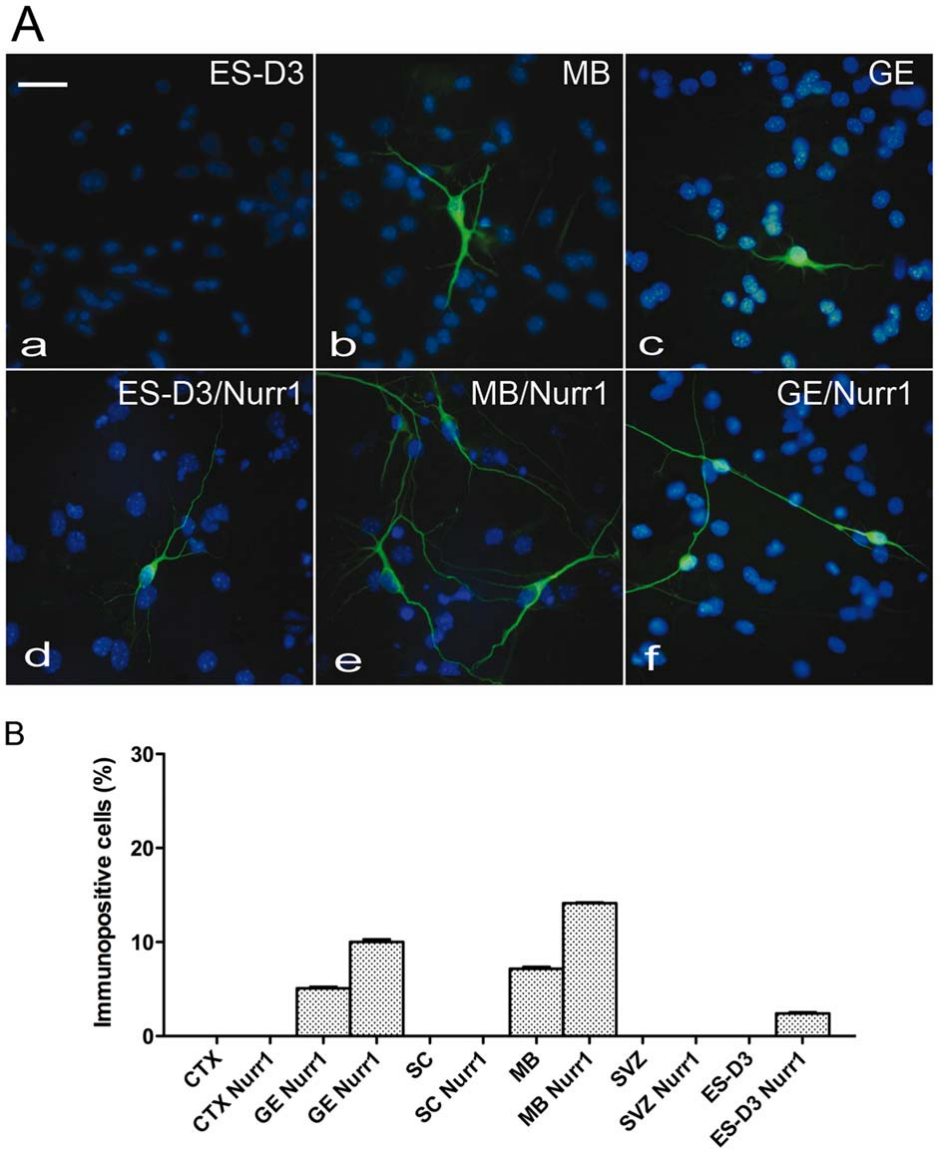
Induction of TH expression by Nurr1 overexpression: immunocytochemistry analysis. A) Immunostaining for TH (green) on differentiated cells in control conditions and after infection with Nurr1 lentivirus. Nuclei were stained with DAPI (blue). (a) ES-D3, (d) Nurr1 infected ES-D3, (b) MB, (e) MB Nurr1 infected (c) GE, (f) Nurr1 infected GE. B) Percentage of TH positive cells (vs total cell number). Scale bar  = 30 μm.

**Figure 6:**
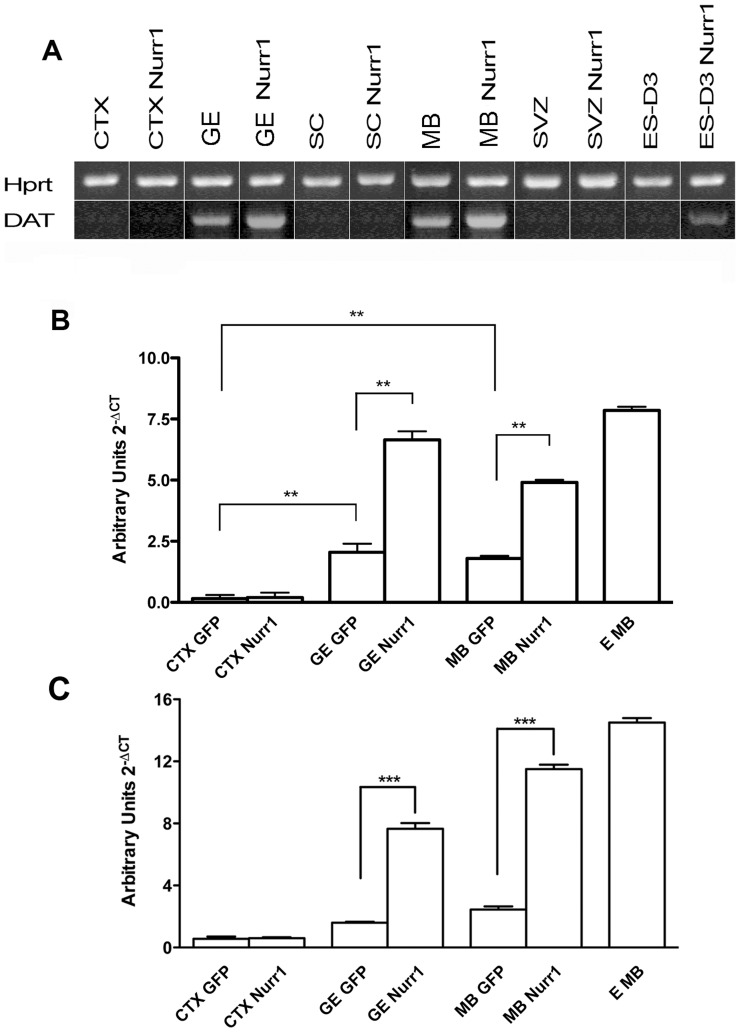
Activation of the dopaminergic markers DAT and Ptx3 by Nurr1 overexpression. A) Representative samples of RT-PCR amplified products for DAT B) and C). real-time PCR of DAT (B) and Ptx3 (C) mRNA on differentiated cells in control conditions and after Nurr1 overexpression HPRT was used as normalizing gene. The error bars represent standard deviation (n = 3). ** P<0,02.

To investigate whether Nurr1 overexpression enhanced dopaminergic marker expression, control and Nurr1-expressing NPs were differentiated and subsequently analysed by RT-PCR and/or Real-time PCR for TH, DAT and Pitx3 expression and immunostained for TH. In these assays, we found that TH mRNA levels was higher in control NPs from GE and MB, compared to control NPs from CTX and SC, consistently with the spatial distribution of DA neurons in vivo. Importantly, compared to control NPs, Nurr1 overexpression caused a significant increase in TH expressiononly in transduced GE- and MB-derived NPs (3–5 fold), but not in CTX- and SC-derived NPs (Fig. 4 and Fig. S1). In addition, Nurr1 also promoted TH expression in NPs derived from mouse ES cells (see below). The presence of TH-positive cells under differentiating conditions in both control and Nurr1-expressing NPs derived from GE and MB, but not from CTX and SC, was revealed by immunostaining (Fig. 5 A). Notably, the number of TH-positive cells was 2-fold higher in Nurr1-overexpressing cultures from GE and MB, when compared to control cultures (Fig. 5 B). Similar results were also obtained with DAT expression (Fig. 6A and B). Previous studies have shown that Nurr1 can directly activate Pitx3 expression and cooperate with Pitx3 in the specification of dopaminergic cell fates [31], [32]. Thus, it was interesting to see that Nurr1 overexpression specifically upregulated Pitx3 in GE- and MB-derived NPs (4–5 fold) and that its expression was not detected in the CTX NPs overexpressing Nurr1 (Fig. 6C). In addition to NPs derived from the embryonic CNS, adult NPs (aNPs) derived from the murine SVZ were subjected to the same experimental procedures. Multipassaged aNPs expressed nestin (Fig. 1 A) and retained the ability to generate both neurons and glial cells (Fig. 3). Nurr1 expression following lentivirus infection yielded overexpression levels comparable to those obtained in embryonic NPs infected with the same vector (Fig. 2). Nonetheless, Nurr1 overexpression was not sufficient to cause formation of TH-positive cells (Fig. 5B) and activate DAT expression (Fig. 6A in aNPs. In conclusion, these results show that, by stage E13.5 of mouse development, Nurr1 is able to promote dopaminergic cell fates specifically in NPs derived from GE and MB, but not in NPs from non-dopaminergic neural areas, such as CTX, SC and adult SVZ.

### ES-D3 cells

As a positive control for our overexpression approaches, we also examined the ability of Nurr1 to promote dopaminergic cell fates in NPs derived from mouse ES cells (ES-D3), as previously described [33], [34]. ES-D3 cells were first induced to differentiate by culturing them as embryoid bodies, followed by selection and expansion of Nestin-positive NPs (see Materials and Methods). As shown in Fig. 1 A, following the differentiation procedure all cells in the culture became Nestin-positive. When NPs derived from ES-D3 cells were infected with Nurr1 lentivirus or GFP lentivirus, we detected by real time PCR a significant increment of Nurr1 mRNA levels 72 hours after infection (Fig. 2). Control (GFP infected cells) and infected ES-D3 were then differentiated upon bFGF deprivation, obtaining about 40% of neurons (beta-III-tubulin-positive cells) and 50% of glial cells (GFAP-positive cells) in both control and infected ES-D3 cells (Fig. 3B). Real-time PCR and immunostaining analyses of infected ES-D3-derived NPs, maintained under differentiating culture conditions, showed a significant increase of TH mRNA levels compared to control cultures, (Fig. 4) and the presence of TH positive cells only in Nurr1-expressing cultures (2–3% of total cells, Fig. 5). Importantly, expression of the dopamine transporter DAT was detected by RT-PCR in Nurr1-infected cells (Fig. 6A), further confirming the induction of the dopaminergic phenotype.

## Discussion

Expandable cells are a useful tool that can be used in transplantation because of the advantage of standardized cell populations, in terms of proliferation and differentiation potential [35]–[38]. In this respect, a question of interest is whether NPs from different brain regions retain the ability to respond to transcription factors involved in the differentiation of specific neuronal populations, once they have acquired a positional identity. To address this issue, we compared the ability of NPs derived from different CNS regions to respond to the transcription factor Nurr1 by activating a dopaminergic phenotype.

NPs from embryonic nervous system and adult neurogenic areas can proliferate in culture in the presence of bFGF and EGF and, under appropriate culture conditions, can generate both neurons and glial cells [39]–[41].

Our results show that NPs derived from E13.5 mouse embryos retain at least in part their positional identity, as shown by preservation of regulated expression profiles of region-specific transcription factors, such as FoxG1, Dlx2, Six3, HoxB6, HoxB9 and Ngn2 and also recently reported for E14.5 NPs [10]. Our data also show that NPs derived from E13.5 mouse embryos respond to Nurr1 overexpression by giving rise to a small number of dopaminergic neurons, but this ability is critically dependent on their area of origin. In fact, Nurr1 overexpression caused an increase of TH mRNA levels and the percentage of TH-positive neurons specifically in GE- and MB-derived NPs, but not in CTX-, SC- or SVZ-derived NPs. Similar effects were detected with other DA-cell markers, such as DAT and Pitx3, indicating that they are not limited to the TH gene but reflect the acquisition of a dopaminergic phenotype. Pitx3 region-specific induction following Nurr1 overexpression is interesting, since Pitx3 expression has been reported to be directly regulated by Nurr1 in mesencephalic cells, due to the presence of a Nurr1 binding region on the Pitx3 promoter [31]. Our results suggest that additional mechanisms modulate the ability of Nurr1 to activate Pitx3 transcription, consistently with the fact that in vivo Pitx3 is expressed only in a subset of Nurr1-positive cells [32]. The ratios of beta-III-tubulin-positive and GFAP-positive cells showed that Nurr1 does not recruit larger numbers of NPs to neurogenic versus gliogenic fates, but rather suggest that the increase of TH-positive cells might be ascribed to NP assignment to a dopaminergic fate. These effects were reproduced with NPs derived from independent dissections and with different experimental approaches to achieve Nurr1 overexpression. Together with the fact that Nurr1 was also able to promote dopaminergic cell fates in ES cells, as previously described [20], these observations strongly argue against the idea that the lack of dopaminergic specification in CTX-, SC- and SVZ-derived NPs in due to insufficient Nurr1 activity in these cells and suggest instead that these differential effects are closely related to the pre-existing regional identity of NPs.

While DA neuronal populations are well known to reside in the ventral midbrain, striatal neurons expressing TH have also been reported in several mammalian species and in humans [42], [43], and the existence of striatal DA neurons, which are similar to MB DA neurons, has also been demonstrated [44]. These observations could explain the fact that Nurr1 is able to promote dopaminergic fates also in GE-derived NPs, besides MB-derived NPs.

Considerable controversy exists concerning the degree to which NPs retain in vitro the positional information acquired in their region of origin. Some studies indicated that NPs maintained their regional specification even after several passages in vitro as neurospheres [5], [9], [45], [46], while others reported a progressive loss of regional identity due to the downregulation of dorsal and ventral markers in culture [29], [47]. It should be mentioned that different properties of NPs depend on the culture conditions used, even at early passages [48], or on the species of origin, e.g. rat [29] or mouse [46]. Armando et al. [45] showed that mouse embryonic CTX, STR and SC derived neurospheres, even after 8 passages, generate in vitro neural progenies that show, after differentiation, region-specifc behaviour. Moreover, NPs derived from human fetal CTX and STR exhibit region-specific differentiation in vitro, survive, migrate, and form mature neurons after intracerebral transplantation in newborn rats [9]. Kim et al. [49] also demonstrated that human neurospheres isolated from different CNS compartments express distinctive molecular markers of regional identity and maintain these patterns of region-specific gene expression during long-term passages in vitro, as also recently reported for mouse cortex NPs [10]. While all these studies have been very informative in showing that some region-dependent traits of NPs can be maintained in vitro, there is limited information available on whether this pre-existing specification can be overcome by forced expression of transcription factors controlling specific cell fates. Our work thus confirms and extends previous studies in this field by showing that NPs cultured in vitro maintain at least some of their original positional information, not only in terms of molecular marker expression or differentiation properties, but, importantly, also in terms of their competence to respond to Nurr1 overexpression by activating a dopaminergic differentiation programme. These results suggest that Nurr1 needs a specific cellular environment and a pattern of gene expression that supports and/or promotes dopaminergic function in some brain regions only and that the presence or absence of this positional signature can be at least partially preserved in vitro. Induction of dopaminergic neurons from human foetal forebrain has been reported also by Christophersen et al. [50]; however in this case TH expression appears to be dependent on the presence of specific factors in the medium, which are required for TH expression. On the other hand, experiments on ES-D3 cells demonstrate that NPs derived from pluripotent uncommitted embryonic ES-D3 can give rise to dopaminergic neurons following Nurr1 overexpression. These results are consistent with previous findings showing the ability of Nurr1 to elicit differentiation of dopaminergic neurons in ES cells [20]–[23]. The higher increase of TH positive cells induced by Nurr1 overexpression in GE and MB NPs compared to the limited increase in ES-D3 cells suggests that the regional specification either prevents (as in CTX, SC and SVZ NPs) or permits/facilitates (as in GE and MB NPs) the activation of a dopaminergic program regulated by Nurr1. The lack of Pitx3 expression in control NPs from midbrain is consistent with the observation that Pitx3 transcripts are present only in committed and differentiated MB dopaminergic neurons [51] and shows that Nurr1 overexpression is not sufficient to turn on this gene, at least in our culture conditions. On the other hand, the increase of TH-positive neurons on MB NPs overexpressing Nurr1, Activation of Pitx3 expression in GE and MB NPs is consistent with the observation that during embryonic development the induction of TH expression in the ventral midbrain requires Nurr1 which in turn activates Pitx3 expression [14]. In conclusion, although interplay between Nurr1 and Pitx3 have been reported to exert a cooperative effect in the induction of MB dopaminergic differentiation in vivo and in vitro [52], [53] Nurr1 overexpression appears sufficient to promote the expression of a subset of midbrain DA neuron markers, such as TH and DAT, possibly via Pitx3 activation.

As far as SVZ derived NPs are concerned, we did not find TH positive neurons from either uninfected or Nurr1 infected adult SVZ cells. On the other hand Shim and colleagues reported that SVZ cells do not generate TH-positive neurons in vitro, even if exposed to cytokines involved in dopaminergic differentiation; however, they obtained functional dopaminergic neurons by overexpressing Nurr1 [54]. The discrepancy with our study could be explained by the fact that Shim's experiments were performed in unpassaged SVZ cells rather than in multipassaged cells. Notably it has been shown that neural precursors from ventral mesencephalon almost completely lose their ability to generate dopaminergic neurons after in vitro expansion [55], which could account for the differences between our and Shim's study. In addition differences between species of origin (rat versus mouse neural precursors) could also account for the different responsiveness to Nurr1, as also observed for maintenance of region specific transcription factor expression [29], [46].

In conclusion we demonstrated that Nurr1 is not sufficient to drive dopaminergic differentiation in all neural precursors, suggesting that cell ability to respond to this transcription factor might depend on the regional specification acquired during early steps of the neural tube regionalization.

## Materials and Methods

### Materials

Cell culture media and fetal calf serum (FCS) were obtained from SIGMA; growth factors (LIF, EGF, bFGF) from R & D Systems; Media Supplements (N2, B27) from Gibco; polyornithine, laminin, fibronectin and other reagents from SIGMA.

### ES cells

The mouse blastocyst-derived ES cell line D3 (ES-D3) obtained from A.T.C.C. (Rockland, MD), was propagated and maintained as previously described [56]. In brief, undifferentiated ES cells were cultured on gelatin-coated dishes in Dulbecco's modified minimal essential medium (DMEM), supplemented with 2 mM glutamine, 0.001% ß-mercaptoethanol, non essential amino acids, 15% fetal bovine serum, and 2000 U/ml human recombinant LIF. Formation of embryoid bodies was obtained upon culture in non adherent bacterial dishes for four days [57]. The resulting embryoid bodies were then plated in adhesive tissue culture dishes. After 24 h in culture, selection for NP cells was initiated in serum-free medium [57]. After 6–10 days of selection, NP were plated on polyornithine (15 mg/ml) and fibronectin (1 mg/ml) coated coverslips in N2 medium supplemented with 1 mg/ml laminin and 10 ng/ml bFGF. After expansion bFGF was removed to induce differentiation.

### Neural Precursors

All animal-related procedures were conducted in accordance with European Communities Council Directive No. 86/609/EEC. Animals were anaesthetized and sacrificed by decapitation.

NPs were obtained from E13.5 mouse embryonic cortex (CTX), spinal cord (SC), midbrain (MB) and ganglionic eminence (GE) and adapted to cell cultures as previously described [26], [58]. Tissues were incubated in Dulbecco's modified Eagle's Medium (DMEM) for 20 min at 37°C and then mechanically dissociated to single cell suspensions by trituration. The cells were transferred to basal medium (DMEM/F12, 1% penicillin/streptomycin, 0.1 M L-glutamine (Gibco), 23.8 mg/100 ml Hepes, 7.5% NaHCO_3_, 0.6% glucose), with the addition of 20 ng/ml human recombinant EGF, 10 ng/ml bFGF, and 1% N2 supplement. This medium was termed embryonic expansion medium. Living cells were counted in the presence of trypan blue and plated in uncoated T25 plastic flasks in embryonic expansion medium. After several days, spontaneous floating clusters were harvested, enzymatically dissociated (Accutase; Sigma) and single cell suspensions were plated in flasks coated with 10 mg/ml poly-ornithine and 5 mg/ml laminin at a density of 0.4–0.6×10^4^ cells/cm^2^; cells between passages 6 and 14 were used. Adult NPs (aNPs) were obtained from the subventricular zone (SVZ) of 2 month-old male mice. Briefly, 1 mm- thick coronal slices containing SVZ were collected in Leibovitz's L-15 medium and the lateral wall of the lateral ventricle was carefully removed from the surrounding brain tissue. SVZ explants were incubated in HBSS (Gibco) containing 5.4 mg/ml D-glucose, 18 mM Hepes, 0.74 mg/ml trypsin, 200 U/ml DNase, 0.7 mg/ml hyaluronidase, 2 mg/ml kynurenic acid (all from Sigma) for 20 min at 37°C and mechanically dissociated by trituration. Living cells were counted in the presence of trypan blue and plated in uncoated plastic flasks. aNPs cells were routinely cultured on poly-ornithine and laminin coated flasks in expansion medium containing B27 instead of N2 (adult expansion medium). Cells between passages 4 and 8 were used. Both embryonic and adult NPs were maintained under proliferative conditions at 37°C in a 5% CO_2_ atmosphere. In order to induce differentiation, cells were cultured for five days in basal medium containing N2, B27 and 10 ng/ml bFGF (bFGF differentiation medium), followed by additional five days omitting bFGF.

### Recombinant lentivirus vectors: virus production and cell infection

To insert Nurr1 in the lentiviral vector pWPXLd, Nurr1 was amplified from CMX Nurr1 plasmid using primers (Up 5′ CTG CAA CGC GTC ATG CCT TGT GTT CAG GCG CAG TAT GGG TCC T-3′; Down 5′- CAG CTA CTA GTC CTT AGA AAG GTA AGG TGT CCA GGA AAA GTT TGT-3′) which contain in the UP primer the digestion site MluI, and in the Down primer the SpeI site. After PCR amplification, the product was cut and inserted into the pWPXLd vector.

Recombinant lentiviruses were derived by the combined transfection of three elements: 1) a transfer vector, 2) the packaging vector pCMV-dR8.74 and 3) the VSV-G envelope vector pMD2G (obtained from D.Trono, http://www.tronolab.com).

To produce recombinant lentiviruses, subconfluent 293T cells plated in 100 mm dishes (around 2.5×10^6^ cells/plate) were cotransfected, according to the manufacturer indications, by means of FuGene 6 (Roche Diagnostics, Indianapolis IN) with the envelope and packaging constructs (5 and 15 µg/dish, respectively) and with the specific transfer construct (20 µg/dish). At 48 and 72 hours post-transfection, supernatants were collected, filtered through 0.45 µm filters, concentrated by ultracentrifugation (90 minutes, 26,000 rpm at 4°C), resuspended in complete medium, aliquoted, and stored at −80°C. Titers of recombinant lentiviruses were determined by p24 antigen quantification as indicated (Alliance HIV-1 p24 ELISA KIT; Perkin-Elmer, Boston, MA). An average value of 142 ng/ml of p24 was obtained from a 10×100 mm dish cotransfection. In all experiments, the multiplicity of infection (MOI) is expressed in picograms p24/cell. As a negative control and to test infection efficiency all cells were infected with lentiviral vectors carrying GFP and analyzed by FACS 72 hours after infection. Accordingly lentivirus carrying Nurr1 was used at MOI 2, which yielded a 26–30% infection efficiency for both ES cells and NPs; higher lentivirus concentrations could not be used as they lead to extensive cell death. Transduction experiments were performed in the presence of 8 µg/ml polybrene, (Sigma). After viral addition, cells were centrifuged at 1,800 rpm, 75 minutes at 32°C, and incubated for 45 minutes at 37°C, under 5% CO_2_. Medium was then replaced and the cells were cultured until further analysis.

### Generation of NP lines with stable Nurr1 expression

Nurr1 open reading frame was amplified by PCR using the following primers containing the MfeI restriction site (Forward primer: 5′-ACT GCA CAA TTG ATG CCT TGT GTT CAG GCG CAGT-3′; Reverse primer: 5′-AGA GCT CAA TTG CTT AGA AAG GTA AGG TGT CCAG-3′) and subcloned into the EcoRI site of the pTP6 expression vector [59]. pTP6 vector contains the CAGG promoter followed by the Nurr1 cDNA and an IRES puromycin resistance gene allowing strong selection for transgene expression. Early passage (p<6) NPs from CTX, GE and SC were transfected with pTP6-Nurr1 with Amaxa Nucleofector and Neural Stem Cell Nucleofector kit (Lonza) according to manufacturer instructions. Two days after transfection, NPs with stable integration of the pTP6-Nurr1 transgene were selected and expanded by adding puromycin (0.66–1 µg/ml) to the culture medium. Control NP lines were generated with stable expression of the previously described pTP6-hrGFP construct [60]. Transgenic NPs were cultured as described above, except that they were maintained in puromycin when grown in proliferating conditions.

### Immunocytochemistry

Cells were fixed for 10 min in 4% para-formaldehyde in PBS, pH 7.4 at room temperature and then washed in PBS containing 1% bovine serum albumin (BSA). Dishes were pre-incubated first in 0.1 M glycine in PBS and then in PBS containing 5% normal rabbit or goat serum and 0.025% Triton X-100 for 30 min at room temperature. Incubation with the primary antibody was carried out overnight at 4°C. Antibodies were diluted in PBS containing 5% normal rabbit or goat serum and 0.025% Triton X-100 as follows: mouse anti-beta-III-tubulin (Promega) diluted 1∶200, mouse anti-nestin (Chemicon) diluted 1∶100, rabbit anti-GFAP (DAKO Cytomation) 1∶200, mouse anti-TH (Chemicon) 1∶200, rabbit anti-TH (Chemicon) 1∶100. Dishes were then washed and incubated for 60 min at room temperature with FITC-conjugated rabbit anti-goat IgG, diluted 1∶100, or TRITC conjugated goat anti-rabbit IgG diluted 1∶100, as secondary antibodies (Jackson). After washing, samples were mounted in DABCO (FLUKA). No staining was observed when primary antibodies were omitted. DNA staining was performed by incubating cells with 1 μg/ml DAPI (SIGMA) in PBS for 10 min at room temperature. Immunofluorescence was observed using either a Zeiss Axiophot microscope or a Leica confocal microscope. Immunostained cells were counted on at least 10 randomly selected microscopic fields of three independent experiments and reported as percentage of DAPI stained cells.

### RNA extraction and RT-PCR analysis

Total RNA was extracted from cell cultures using the Trizol extraction procedure (Invitrogen) and digested with DNAse I (Ambion). For each RNA sample, 2 µg of total RNA were reverse transcribed for 60 min at 37°C with random hexamers (Promega) as primers and M-MLV reverse transcriptase (Promega). A 2 µg aliquot was treated identically except that reverse transcriptase was omitted. A control was also included containing all reagents (including reverse transcriptase) except RNA. After reverse transcription, GoTaq DNA Polymerase (Promega) primers (final concentration 0.4 µM each primer) and the appropriate buffer were added (final volume 25 µ l) to the tubes. PCR conditions were as follows: initial denaturation at 95°C for 2 min, followed by 30 cycles with the following profile: 95°C for 30–45 sec, 55°C for 30–45 sec, 72°C for 30–60 sec, final extension at 72°C for 5 min. 25 µl of each PCR reaction product were run on a 0.8% agarose gel containing 1 µ g/ml ethidium bromide to reveal DNA bands (**Primers sequence are available under request**).

### Real-Time/RT-PCR and quantification of gene expression

Real-time PCR amplifications were performed on reverse transcription (RT) products with the SYBR Green JumpStart Taq ReadyMix (Qiagen, Valencia, CA, USA) in a Lightcycler apparatus (Bio-Rad, Hercules, CA, USA), following the manufacturer's instructions. All samples were run in duplicate, and each well contained 25 µl as a final volume, including 2.5 µl of cDNA, 0.2 µM forward primer, 0.2 µM reverse primer, and 12.5 µl SYBR Green JumpStart Taq ReadyMix and 0.2 µl internal reference dye. The PCR started with 94°C for 2 min and then continued with 35–40 cycles of 30 s at 94°C, 30 s at 60°C, 45 s at 72°C, and 15 s at 80°C. A melting curve was obtained for each PCR product after each run to confirm that the signal corresponded to a unique amplicon of the predicted size. The specificity of the PCR product was verified by sequencing. Expression levels were normalized to the housekeeping genes encoding for Actin, HPRT or PBGD, obtained for every sample in parallel assays of two to four independent experiments. The mRNA levels for each gene were compared between cells infected with either the Nurr1 containing lentivirus vector or the corresponding empty vector by using the DDCT method (Primers sequence are available under request).

## Supporting Information

Figure S1 **Nurr1 and TH expression in in NP lines with stable expression of Nurr1.** NP lines were selected for stable expression of Nurr1 as described in Materials and Methods. A and B. real-time PCR of Nurr1 (A) and TH mRNA (B) on differentiated cells in control conditions after Nurr1 overexpression, HPRT was used as normalizing gene. C. Percentage of beta-III-tubulin and GFAP positive cells after differentiation of NPs. The error bars represent standard deviation (n = 3). ** P<0.02.
(TIF)
