# Supplementary figures and images for: Restriction of Neural Precursor Ability to Respond to Nurr1 by Early Regional Specification
Source: PLoS One. 2012 Dec 11;7(12):e51798. doi: 10.1371/journal.pone.0051798 (PMC3519900; doi:10.1371/journal.pone.0051798)

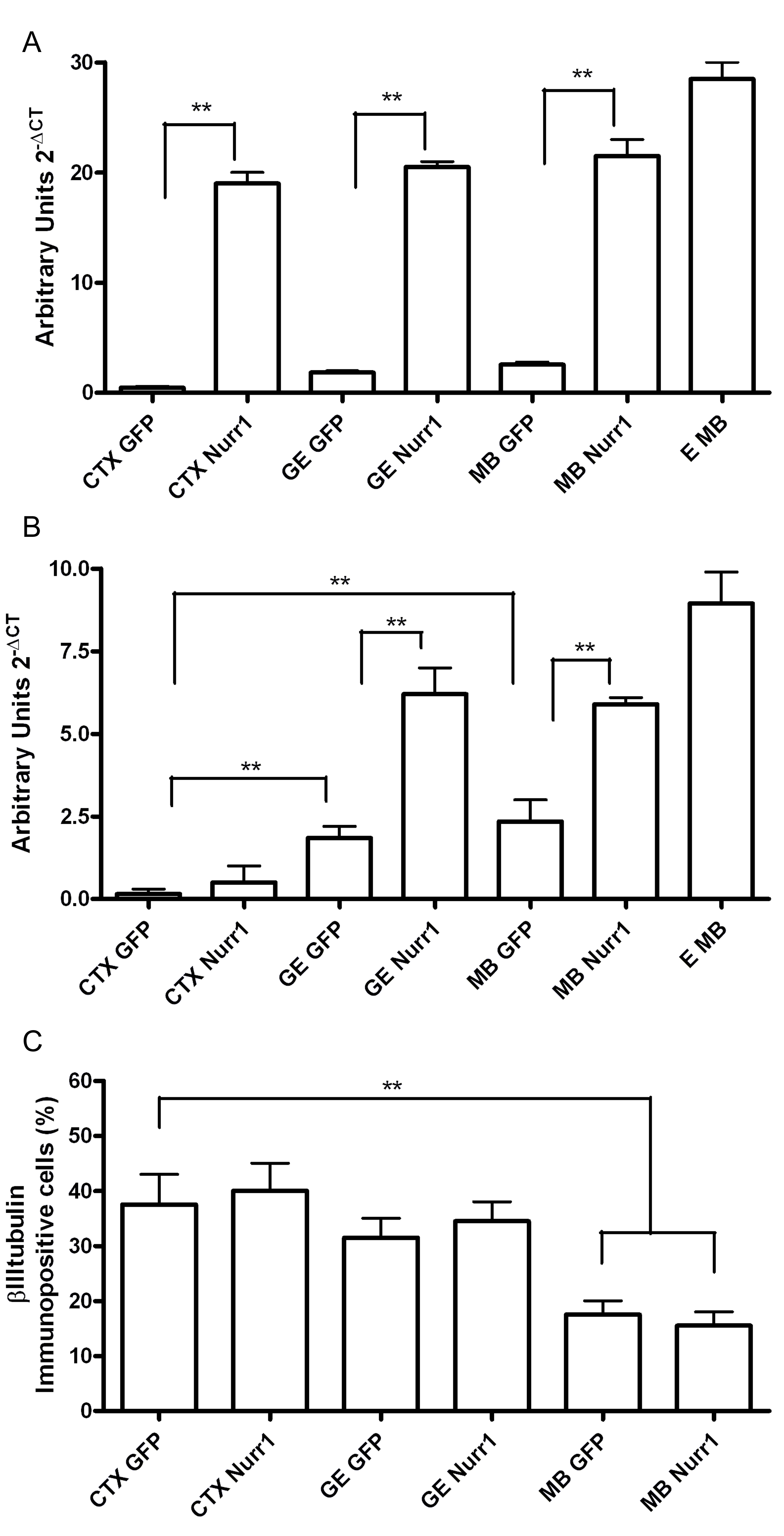

Supplement: Figure S1 — Nurr1 and TH expression in in NP lines with stable expression of Nurr1. NP lines were selected for stable expression of Nurr1 as described in Materials and Methods. A and B. real-time PCR of Nurr1 (A) and TH mRNA (B) on differentiated cells in control conditions after Nurr1 overexpression, HPRT was used as normalizing gene. C. Percentage of beta-III-tubulin and GFAP positive cells after differentiation of NPs. The error bars represent standard deviation (n = 3). ** P<0.02. (TIF) [file pone.0051798.s001.tif]
